# Editorial: State-of-the-Art Research on C1q and the Classical Complement Pathway

**DOI:** 10.3389/fimmu.2016.00398

**Published:** 2016-10-04

**Authors:** Uday Kishore, Nicole M. Thielens, Christine Gaboriaud

**Affiliations:** ^1^College of Health and Life Sciences, Brunel University London, Uxbridge, UK; ^2^Institut de Biologie Structurale (IBS), Université Grenoble Alpes, CEA, CNRS, Grenoble, France

**Keywords:** complement, C1 complex, C1q, gC1q structure, C1s, classical pathway activation, autoimmunity, emerging functions

**The Editorial on the Research Topic**

**State-of-the-Art Research on C1q and the Classical Complement Pathway**

Complement protein C1q is a fascinating innate immune molecule. C1q is the first subcomponent of the classical complement pathway. Its primary three-chain structure (A, B, and C chains), which is composed of a triple-helical collagen-like region and a C-terminal ligand-recognizing globular head (gC1q) domain, yields a tulip-like organization with six gC1q domains, each representing a heterotrimer (the C-terminal regions of the A, B, and C chains) (1). In addition to binding to immune complexes containing IgG and IgM, the gC1q domain also engages with a number of self and non-self ligands. A summary of the structural basis of C1q–ligand interactions has been elegantly presented in the review by Gaboriaud et al. For example, it shows that C1q can recognize multiple ligands on the apoptotic cell surface, which illustrates its versatile surface recognition properties. A common binding area for several of these non-immune ligands has been observed in the subunit C, which could play a role in restricting the activation of the classical complement pathway.

It has become evident over the course of the last two decades that the gC1q domain is widely conserved across a diverse range of vertebrate and invertebrate proteins (2). The functions of these proteins can range from being immunological to structural. Through a review article, Ghebrehiwet et al. have cited two examples of C1q functions that are distinct from its involvement in the classical pathway: its ability to induce apoptosis in prostate cancer cells, and to modulate vascularization for fetal–maternal interaction. Given the existence of a C1q–TNF superfamily based on the remarkable structural similarities between C1q and TNF family members (1), a good argument has been made for a cytokine-like property of C1q. The complexity and diversity related to the functions of C1q family have been greatly exemplified by a structure–function review article by Colombatti et al., who describe the functions associated with the gC1q domain of two C1q family members – such as EMILIN and multimerin. A slight structural variation from the prototypical structure, as revealed by the NMR solution structure, is insightful, purporting one residue in the interaction of gC1q domain with α_4_β_1_ and α_9_β_1_ integrins.

In recent years, a number of functions of C1q have emerged that are complement independent. This is reflected in the local synthesis of C1q by various immune and non-immune cells. The diversity of C1q functions includes its involvement in dendritic cell maturation, immune modulation, cell differentiation, cancer progression, neuronal synapse pruning, and pregnancy. This has been extensively summarized by Kouser et al. In the backdrop of a recent observation that C1q gene knockout mice show nearly all features of preeclampsia, the role of C1q in normal and complicated pregnancies has become a burning issue in the reproductive immunology. In this issue, Madhukaran et al. have reported a link between the expression of the transcription factor PU.1 and C1q in human trophoblasts and stromal cells, similar to DCs and macrophages, using early decidual tissue.

Given its involvement in a wide range of homeostatic functions, it is not surprising that C1q is central to many human diseases. C1q, as a key molecule in self-tolerance mechanisms, is involved in clearing immune complexes and apoptotic/necrotic cells. C1q-deficient mice have been shown to have lupus-like symptoms. However, this issue is further confounded by the fact that anti-C1q autoantibodies are found in a number of pathological situations, more so in systemic lupus erythematosus (SLE). These autoantibodies are certainly required for the development of lupus nephritis. Since C1q itself is an IgG-binding protein, identification and characterization of anti-C1q autoantibodies is a challenging task. Mahler et al. have addressed the technicalities of this endeavor, while reassessing the pathological consequences of such autoantibodies in a disease context. In line with the involvement of C1q in tolerance and SLE, Ghebrehiwet et al. have addressed the importance of monocyte surface expressed C1q in association with C1r and C1s. This review examines the role of cell-bound C1q in capturing and processing circulating immune complexes and pathogen-associated molecular patterns (PAMPs). The ability of C1q to modulate PAMP-recognizing receptors (PRRs) makes it a potent and versatile immune surveillance molecule of the innate immunity.

The final section of the issue is dedicated to the understanding of the mechanisms underlying activation of the classical pathway and their specific inhibition for therapeutic purposes.

In an original research article, Wijeyewickrema et al. aim at further deciphering the interaction of the C1s protease with its C4 protein substrate. Previous studies had provided evidence for homologous C4 interaction exosites in the CCP and serine protease domains of both C1s and MASP-2 (3). The authors focus here on the role of C1s Lys628 residue of the serine protease domain. Using site-directed mutagenesis and a peptide substrate library, they show that this residue plays a different role in cleaving peptide versus protein substrates by interacting with C4 in order to facilitate its cleavage. The architecture of the active site at this position is markedly different in C1s compared with MASP-2 (4), which might provide clues toward designing specific inhibitors of the classical pathway.

In a review article, Sharp et al. focus on complement inhibitors of the classical pathway for application in transfusion medicine. Uncontrolled antibody-initiated complement activation plays a central role in hemolytic diseases such as acute intravascular hemolytic transfusion reaction (AIHTR). The authors have identified peptide inhibitors of C1 (PIC1) derived from a region of the coat protein of astrovirus with homology to human neutrophil peptide 1 that inhibits complement activation by binding to the collagen-like regions of C1q (5). They have developed a simple rat model of AIHTR for future preclinical studies of PIC1. A review of complement inhibitors, marketed as well as currently under development, is presented, and their therapeutic potential in transfusion and blood disorders is discussed.

In their perspective article, Gaboriaud et al. provide an update on the current knowledge about the structural basis of the mechanisms involved in the assembly and activation of the C1 complex. The main protein players involved in C1 activation and its control are presented together with the molecular dissection strategy used to define structure–function relationships of C1 subcomponents and to decipher key protein–protein interactions. However, the conformational changes required for allowing C1 activation following target binding are still not elucidated, and this is one among 16 still unanswered questions identified by the authors. Hopefully, new technological developments in structural biology, such as a combination of X-ray crystallography and electron microscopy, will help fulfilling this “mission impossible.”

This special topic issues clearly highlights the diversity and complexity associated with structures and functions of C1q and C1q family members. We have come a long way in understanding the structural basis of the gC1q interaction with self and non-self ligands. While additional members of C1q family are still being identified and characterized, the importance of the gC1q domain in the evolutionary history of animals is unique. From being a prototypical innate immune molecule, C1q is now regarded as an independent modulator of a diverse range of functions that are not dependent on its involvement in the complement activation. These complement-independent functions of C1q are likely to be the major thrust of research in coming years.

## Author Contributions

All authors listed have made substantial, direct, and intellectual contribution to the work and approved it for publication.

## Conflict of Interest Statement

The authors declare that the research was conducted in the absence of any commercial or financial relationships that could be construed as a potential conflict of interest.
